# Field-testing the explicit diagnostic criteria for transient ischemic attack: a diagnostic accuracy study

**DOI:** 10.1007/s00415-024-12733-2

**Published:** 2024-12-16

**Authors:** Adrian Scutelnic, Seraina Michèle auf der Maur, Mattia Branca, Morin Beyeler, Thomas Horvath, Philip Bücke, Thomas Meinel, Elena R. Lebedeva, Jes Olesen, Franz Riederer, Tomas Dobrocky, Jan Gralla, Marcel Arnold, Urs Fischer, Heinrich P. Mattle, Simon Jung, Christoph J. Schankin

**Affiliations:** 1 Department of Neurology, Inselspital, University Hospital Bern, University of Bern, Freiburgstrasse, 3010 Bern, Switzerland; 2 CTU Bern, University of Bern, Bern, Switzerland; 3 Department of Neurology, The Ural State Medical University, Yekaterinburg, Russia; 4 International Headache Center “Europe-Asia”, Yekaterinburg, Russia; 5 Danish Headache Center, Department of Neurology, Glostrup Hospital, University of Copenhagen, Copenhagen, Denmark; 6 Institute of Diagnostic and Interventional Neuroradiology, Bern University Hospital, Bern, Switzerland

**Keywords:** Migraine aura, ischemic stroke, transient ischemic attack, score, accuracy

## Abstract

**Background and aim:**

Explicit diagnostic criteria for transient ischemic attack (TIA) (EDCT) have been recently proposed based on the assumption, that a migraine aura-like symptom is not typical for a TIA. However, migraine-like symptoms have been unexpectedly frequent in patients with confirmed ischemic stroke. This cross-sectional study aimed to field-test the EDCT to distinguish transient neurological symptoms caused by cerebral infarction from those caused by migraine aura.

**Methods:**

The sensitivity, specificity, positive and negative predictive values of the EDCT score were calculated in samples of patients with (i) transient symptoms caused by cerebral infarction confirmed by imaging and (ii) patients with migraine with aura diagnosed according to the International Classification of Headache Disorders 3rd edition. Sensitivity, specificity, positive and negative predictive values of the original and modified EDCT were calculated, as well as area under the curve adjusted for age and sex using the logistic regression method.

**Results:**

The study population included 59 patients with cerebral infarction and 324 patients with migraine with aura. The median age of the stroke group was 72 (IQR 61–81) and of the migraine group 39 (IQR 29–53). There were 36 (61%) men in the stroke group and 221 (68%) women in the migraine group. For the detection of TIA with imaging-proven cerebral infarction, the *original* EDCT had a sensitivity of 90% (95%CI 79–96), a specificity 77% (95%CI 72–82), a positive predictive value of 42% (95%CI 33–51), and the negative predictive value 98% (95% CI 95–99). For the *modified* EDCT, the sensitivity was 81% (95%CI 69–90), the specificity 97% (95%CI 94–98), the positive predictive value 81% (95%CI 69–90), and the negative predictive value 97% (95%CI 94–98).

**Conclusions:**

The original and modified EDCT criteria miss up to 1 of 10 and 1 of 5 patients, respectively, with transient symptoms due to cerebral infarction. However, the modified EDCT criteria are more specific but less sensitive in detection of ischemic events. The optimal combination of clinical markers to reliably distinguish TIA from migraine aura remains to be found.

**Supplementary Information:**

The online version contains supplementary material available at 10.1007/s00415-024-12733-2.

## Introduction

Currently, there is no diagnostic test that is widely available, sensitive and specific enough for the diagnosis or exclusion of a transient ischemic attack (TIA) and the diagnosis often remains uncertain in clinical practice. The currently endorsed American Heart Association/American Stroke Association (AHA/ASA) definition specifies that a TIA should cause reversible symptoms without evidence of brain infarction on imaging [1]. However, what symptoms a TIA can have has not been specified by the current definitions. The proposal of diagnostic criteria for TIA called Explicit Diagnostic Criteria for Transient Ischemic Attack (EDCT) [2] is an attempt based on the assumption that they are opposite to those of migraine aura. A modified version excluded migraine-like irritative as well as gradually or successively occurring symptoms from the clinical spectrum of the TIA [3]. These criteria reflected previously published clinical features of TIA, such as sudden onset of symptoms and usually negative symptoms consisting of typically hemiparesis, hemihypesthesia, aphasia, amaurosis fugax, hemianopsia or hemiataxia [4–6]. As in ischemic stroke, a TIA is caused by a decreased blood flow to the brain, leading to loss of function of affected areas of the brain. Conversely, a migraine aura usually consists of irritative symptoms, which are required by the current diagnostic criteria [7].

Recently, migraine-like symptoms have been reported in patients with confirmed ischemic stroke [8]. Possible mechanisms of irritative symptoms in stroke include ischemia-induced cortical spreading depression, thrombus migration, gradual collateral-failing and cortical disinhibition phenomena [9–11].

Here, we field-tested the original and modified EDCT with a focus on misclassification of ischemic events as non-ischemic. The aim of our study was to assess the performance of the original and modified EDCT to distinguish if transient neurological symptoms are due to acute ischemic events or caused by migraine with aura.

## Methods

This paper reports a subgroup analysis of a larger cohort study investigating the clinical presentation of ischemic stroke and migraine aura by interviewing prospective enrolled patients based on a structured questionnaire (supplemental file), at bedside or by phone [8]. Among others, the questions covered the type of symptoms, the duration of symptom onset and the headache characteristics. During the interview, the questions were asked in an understandable language to minimize the risk of misinterpretation.

**Fig. 1:**
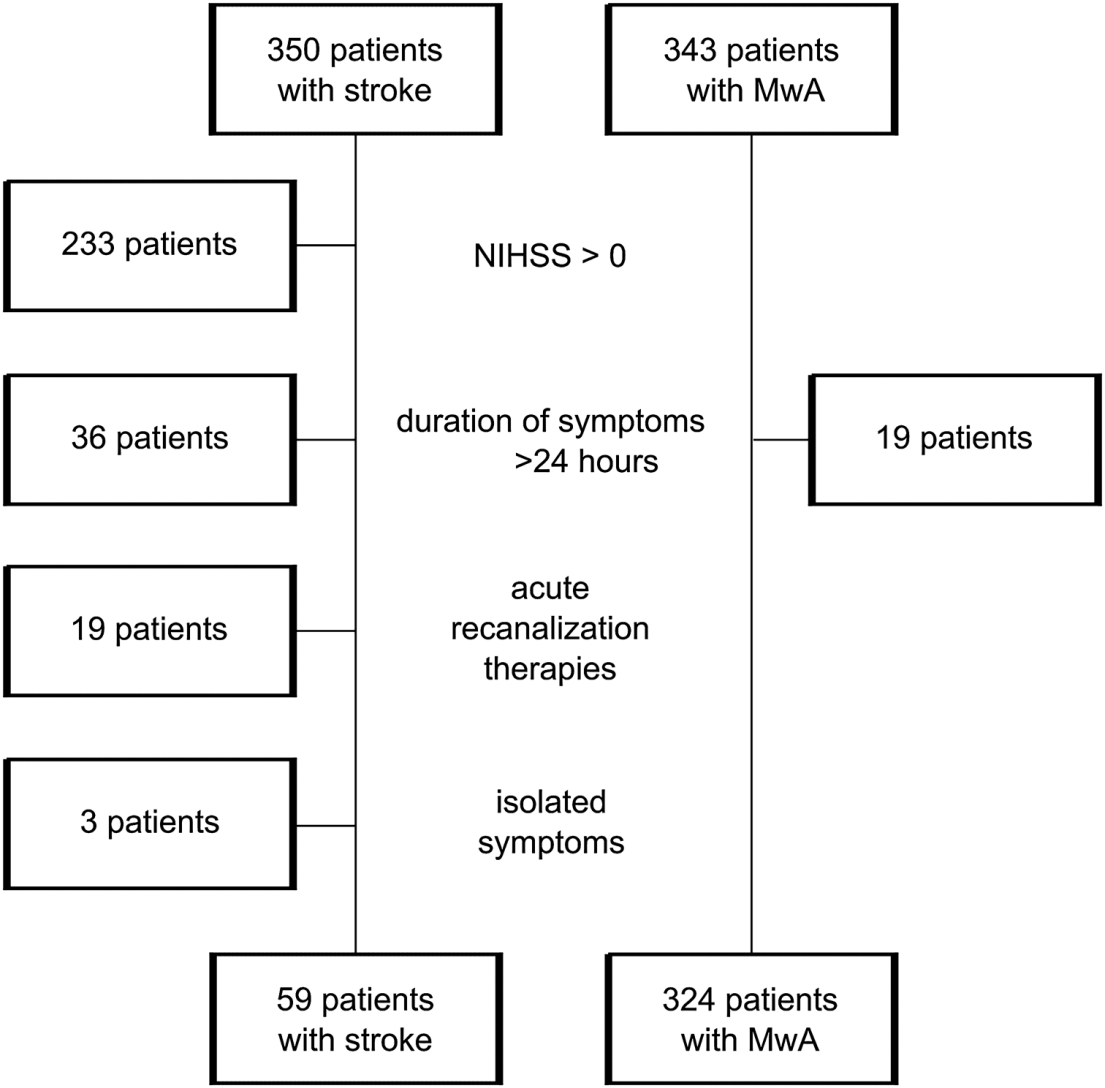
Populations investigated in this accuracy study with the main exclusion criteria: persistent neurological deficit, symptoms lasting longer than 24 hours, acute stroke therapy, and isolated symptoms not compatible with migraine aura

Patients with ischemic stroke confirmed by imaging were included when they had transient symptoms, i.e. when they would, based on clinical judgement alone without performing imaging, be diagnosed with TIA. These patients were classified as having cerebral infarction with transient symptoms [1]. The following exclusion criteria were applied (Fig. 1): (i) persistent neurological deficit (i.e. NIHSS > 0), (ii) symptoms lasting longer than 24 h, (iii) reversible symptoms with acute therapy such as intravenous thrombolysis or endovascular therapy, (iv) isolated symptoms not compatible with migraine aura, such as shaking spells, diplopia, dizziness, vertigo, syncope, decreased level of consciousness, confusion, hyperventilation associated paresthesia, unexplained falls, or amnesia. The initial diagnosis of stroke was made by board certified neurologists on the emergency department.

For testing the EDCT criteria, we included patients with migraine with aura (MwA). The inclusion criteria for the participants with MwA were: (i) diagnosis of definite MwA according to ICHD-3 [7], (ii) last migraine attack with aura within 12 months prior to study inclusion, and (iii) age ≥ 18 years. For the migraine aura group, we excluded patients with symptoms lasting longer than 24 h. The final adjudication of cases with regard to the final diagnosis of MwA and stroke was done by board certified neurologists (AS, CJS).

### Statistical analysis

The statistical analysis was done in STATA/MP 16.0, Statacorp LCC. Categorical variables are presented in counts and non-normally distributed continuous variables in means and interquartile ranges. We calculated the sensitivity, specificity, positive and negative predictive values of the original and modified EDCT (Table 1) based on the data obtained from both groups. In addition, we calculated the optimal cut-off value with the corresponding sensitivity and specificity using the Youden method.

**Table 1:** The explicit diagnostic criteria for transient ischemic attacks (EDCT) [2, 3]

A Sudden onset of fully reversible neurological or retinal symptoms (typically hemiparesis, hemihypesthesia, aphasia, neglect, amaurosis fugax, hemianopsia, or hemiataxia)
B Duration < 24 h
C At least two of the following
C1 At least 1 Symptom is maximal in < 1 min (no gradual spread)
C2 2 or more symptoms occur simultaneously
C3 Symptoms in the form of deficits (no irritative symptoms such as photopsias, pins and needles, etc.)
C4 No headache accompanies or follows the neurological symptoms within 1 h
C* At least two of the following
C1* All symptoms are maximal in < 1 min (no gradual spread)
C2* All symptoms occur simultaneously
C3* All symptoms are deficits (no irritative symptoms such as photopsias, pins and needles, etc.)
C4* No headache accompanies or follows the neurological symptoms within 1 h
D None of the following isolated symptoms (can occur together with more typical symptoms): shaking spells, diplopia, dizziness, vertigo, syncope, decreased level of consciousness, confusion, hyperventilation associated paresthesias, unexplained falls or amnesia
E No evidence of acute infarction in the relevant area on neuroimaging

The C criterion is different between the original and the modified* version

Because we included patients with ischemic lesions only, the E criterion of the EDCT was not fulfilled in any stroke patients.

Demographic characteristics such as age may influence the type of symptoms during ischemic stroke and migraine aura [12, 13]. Therefore, we calculated the area under the curve (AUC) of the receiver operator characteristic controlled for age and sex using the logistic regression method.

Migraine aura of sudden onset might pose an important differential diagnosis in clinical practice. As a sensitivity analysis, we, therefore, compared the fulfilment of EDCT criteria of a migraine aura group, when patients had at least one symptom with onset faster than 1 min, to the stroke group. As a further sensitivity analysis, we compared the fulfilment of EDCT criteria after including MwA patients with interview < 7 days prior to the last migraine aura attack to reduce the influence of recall bias. Recurrent TIA is associated with a higher risk of ischemic stroke [14]. Therefore, we performed a sensitivity analysis by including patients with recurrent symptoms only.

### Standard protocol approvals, registrations, and patient consents

This study was approved by the cantonal ethics committee of canton Bern (KEK 2018-02258). Written informed consent was obtained from all participants. This manuscript adheres to the STROBE guidelines for observational cohort studies.

## Results

Patients were included between 03/2019 and 08/2021. Of the 350 patients with ischemic stroke and 343 patients with MwA, 59 patients and 324 patients, respectively, were included in the analysis. Of stroke patients, 117/350 (33%) had resolved symptoms at the time of admission (Fig. 1). The median age of the stroke group was 72 (IQR 61–81) and of the migraine group 39 (IQR 29–53). There were 36 (61%) men in the stroke group and 221 (68%) women in the migraine group (Table 2). The patients in the stroke group were interviewed a median of 1 day (IQR 1–4) after the ischemic event; the migraine patients a median of 22 days (IQR 7–101) after the last migraine aura attack. Patients with MwA had experienced a median of 53 (IQR 10–288) auras prior to the interview.

**Table 2:** Baseline characteristics of the study population

	Stroke (*N* = 59)	Migraine with aura (*N* = 324)
Age, y, median (IQR)	72 (61–81)	39 (29–53)
Female sex, *n* (%)	23 (39)	221 (68)
Smoking, *n* (%)	16 (27)	97 (30)
Illicit drugs, *n* (%)	0	12 (4)
High blood pressure, *n* (%)	37 (63)	63 (19)
Diabetes mellitus, *n* (%)	12 (20)	8 (3)
Dyslipidemia, *n* (%)	50 (84)	28 (9)
Sleep apnea, *n* (%)	27 (45)	13 (4)
Family history of stroke, *n* (%)	17 (29)	74 (23)
Depression, *n* (%)	12 (20)	82 (25)
Hormonal contraception, women only, *n* (%)	0	34 (15)
Chronic renal failure, *n* (%)	9 (15)	1 (0.3)
Chronic inflammatory disease or cancer, *n* (%)	10 (17)	23 (7)

The distribution of the EDCT criteria for both groups is shown in Table 3.

**Table 3:** The original and modified* explicit diagnostic criteria for transient ischemic attacks (EDCT) in cerebral infarction with transient symptoms and migraine with aura

EDCT	Stroke (*N* = 59)	Migraine with aura (*N* = 324)
A Sudden onset of fully reversible neurological or retinal symptoms (typically hemiparesis, hemihypesthesia, aphasia, neglect, amaurosis fugax, hemianopsia, or hemiataxia), *n* (%)	50 (85)	67 (21)
B Duration < 24 h, *n* (%)	59 (100)	324(100)
C At least two of the following, *n* (%)	53 (90)	74 (23)
C1 At least 1 symptom is maximal in < 1 min (no gradual spread), *n* (%)	50 (85)	67 (21)
C2 2 or more symptoms occur simultaneously, *n* (%)	14 (24)	30 (9)
C3 Symptoms in the form of deficits (no irritative symptoms such as photopsias, pins and needles, etc.), *n* (%)	42 (71)	48 (15)
C4 No headache accompanies or follows the neurological symptoms within 1 h, *n* (%)	40 (68)	16 (5)
C* At least two of the following, *n* (%)	48 (81)	11 (3)
C1* All symptoms are maximal in < 1 min (no gradual spread), *n* (%)	42 (71)	9 (3)
C2* All symptoms occur simultaneously, *n* (%)	9 (15)	2 (1)
C3* All symptoms are deficits (no irritative symptoms such as photopsias, pins and needles, etc.), *n* (%)	39 (66)	3 (1)
C4* No headache accompanies or follows the neurological symptoms within 1 h, *n* (%)	40 (68)	16 (5)
D None of the following isolated symptoms (can occur together with more typical symptoms): shaking spells, diplopia, dizziness, vertigo, syncope, decreased level of consciousness, confusion, hyperventilation associated paresthesias, unexplained falls or amnesia, *n* (%)	59 (100)	324 (100)
E No evidence of acute infarction in the relevant area on neuroimaging, *n* (%)	0	n/a

The sensitivity of the original EDCT for the diagnosis of cerebral infarction with transient symptoms was 90% (95%CI 79–96), the specificity 77% (95%CI 72–82), the positive predictive value 42% (95%CI 33–51), and the negative predictive value 98% (95% CI 95–99).

The sensitivity of the modified EDCT for the diagnosis of cerebral infarction with transient symptoms was 81% (95%CI 69–90), the specificity 97% (95%CI 94–98), the positive predictive value 81% (95%CI 69–90), and the negative predictive value 97% (95%CI 94–98).

For the optimal cut-off value of the original EDCT, Youden index was 0.67, and the area under the ROC curve was 0.83, with a sensitivity at cutpoint of 90% and a specificity of 77%. For the modified EDCT, Youden index was 0.78, and the area under the ROC curve was 0.89, with a sensitivity at cutpoint of 81% and a specificity of 97%.

### Sensitivity analyses

For the entire cohort, the crude AUC and the AUC controlled for age and sex are given in Table 4**.**

**Table 4:** The area under the curve

	Unadjusted area under the curve	For age and sex adjusted area under the curve
Original EDCT	0.69	0.71
Modified EDCT	0.89	0.93
Original EDCT^a^	0.5	0.73
Modified EDCT^a^	0.83	0.91

^a^Tested against migraine with aura with at least one symptom with onset < 1 min

Looking at the subgroup of migraine patients who had at least one symptom with onset faster than 1 min (*n* = 67, 21%), all patients fulfilled the original EDCT. The modified EDCT were fulfilled in 10/67 (15%) migraine patients. The sensitivity of the original EDCT was 90% (95%CI 79–96), the specificity 0% (95%CI 0–5), the positive predictive value 44% (95%CI 35–54) and the negative predictive value 0% (95%CI 0–46). The sensitivity of the modified EDCT was 81% (95%CI 69–90), the specificity 85% (95%CI 74–93), the positive predictive value 83% (95%CI 71–91) and the negative predictive value 84% (95%CI 73–92).

After including migraine patients with interview < 7 days prior to the last migraine attack (94/324, 29%) only, the sensitivity of the original EDCT was 90% (95%CI 79–96), specificity 81% (95%CI 71–88), the positive predictive value 75% (95%CI 63–84) and the negative predictive value 93% (95%CI 85–97). The sensitivity of the modified EDCT was 81% (95%CI 69–90), the specificity 98% (95%CI 93–100), the positive predictive value 96% (95%CI 86–100) and the negative predictive value 89% (95%CI 82–95).

After including stroke patients with recurrent symptoms only (38/59, 64%), the original EDCT had a sensitivity of 89% (95%CI 75–97), a specificity of 77% (95%CI 72–82), a predictive positive value of 31% (95%CI 23–41) and a NPV of 98% (95%CI 96–100). For the modified EDCT the values were as follows: sensitivity 82% (95%CI 66–92), specificity 97% (95%CI 94–98), positive predictive value 74% (95%CI 56–86) and negative predictive value 98% (96–99).

Characteristics of the stroke group are given in Table 5 and characteristics of stroke patients not fulfilling the EDCT criteria in Supplemental Table 1.

**Table 5:** Symptoms, imaging findings and etiology of stroke

Baseline characteristics	Stroke (N = 59)
Symptoms^a^	
Visual, n/N (%)	15/59 (25)
Positive symptoms, n/N (%)	6/59 (10)
Negative symptoms, n/N (%)	12/59 (20)
Sensory, n/N (%)	26/59 (44)
Positive symptoms, n/N (%)	17/59 (29)
Negative symptoms, n/N (%)	16/59 (27)
Motor, n/N (%)	23/59 (39)
Speech, n/N (%)	31/59 (53)
Multiple symptoms, n/N (%)	27/59 (46)
Consecutive, n/N (%)	14/59 (24)
Headache at the time of stroke, n/N (%)	19/59 (32)
Imaging modality	
Magnetic resonance imaging, n/N (%)	56/59 (95)
Computed tomography, n/N (%)	3/59 (5)
Stroke location^a^	
Anterior cerebral artery, n/N (%)	3/59 (5)
Middle cerebral artery, n/N (%)	38/59 (64)
Posterior cerebral artery, n/N (%)	7/59 (12)
Vertebrobasilar^b^, n/N (%)	19/59 (32)
Stroke etiology	
Cardiac embolism, n/N (%)	11/59 (20)
Large artery atherosclerosis, n/N (%)	14/59 (24)
Cervical artery dissection, n/N (%)	1/59 (2)
Patent foramen ovale, n/N (%)	3/59 (5)
More than one possible etiology, n/N (%)	7/59 (12)
Other etiology, n/N (%)	4/59 (7)
Unknown etiology despite complete evaluation, n/N (%)	3/59 (5)
Unknown etiology with incomplete evaluation, n/N (%)	12/59 (23)

^a^Multiple possible

^b^Vertebrobasilar includes brainstem and cerebellum

## Discussion

The main findings of our study are: In people with cerebral infarction with transient symptoms, the explicit diagnostic criteria for transient ischemic attacks (EDCT) in its original and modified version are not fulfilled in 1 of 10 and 1 of 5 patients, respectively. The modified EDCT criteria have a high specificity for differentiating an ischemic event from a migraine with typical aura. When testing against migraine aura with atypical features, such as rapid symptom onset < 1 min, the specificity of the EDCT criteria was low while the modified criteria retained a high specificity.

Our study included patients with focal transient symptoms being, prior to the imaging results, clinically identical to TIA. The confirmation of ischemia in imaging, however, leaves no doubt on the ischemic origin of the symptoms. This is in contrast to previous studies investigating TIA symptoms [2, 3], which had a potential bias of erroneous assignment of patients with strokes to the migraine group and vice versa. On the other hand, since we did not study patients with TIA without ischemic lesion on imaging, we cannot easily transfer our interpretation to this group.

Putting the EDCT into current and historical perspective [15], they significantly improve the clinical differentiation between ischemic and non-ischemic origin of transient neurological symptoms. However, based on our data, they might misclassify up to 10–20% of cases with transient symptoms as non-ischemic although they were caused by cerebral ischemia. This might be due to the exclusion of migraine-like symptoms from the clinical spectrum of ischemic brain events in the development and validation of these criteria [7]. Migraine-like gradual and irritative symptoms in ischemic events have been previously reported [16–19]. The EDCT in its current versions do not account for such symptoms, which might result in the risk to misdiagnose TIA as MwA [20].

We found a very high specificity of the modified EDCT criteria (97%). This means that patients not fulfilling these criteria very likely do not have brain ischemia. This also means, that purely negative symptoms are less likely to occur in patients with definite MwA, since the ICHD-3 criteria require at least one positive symptom with a slow onset [7]. Therefore, testing the modified EDCT criteria against a population with definite MwA diagnosed according to the ICHD-3 criteria would, unsurprisingly, lead to a high specificity. However, the difficult clinical situation is the differentiation of MwA with atypical features (i.e. probable MwA [7]) from a TIA. For this, the applicability of the original and modified EDCT criteria is uncertain. Nevertheless, the AUC for the modified EDCT tested against migraine aura with at least one atypical feature (i.e. symptom onset < 1 min) showed good diagnostic accuracy (AUC 0.83, aAUC 0.91).

In contrast to the original study, we found a lower specificity of the original EDCT criteria in our population (77% in our study vs 95% in the development study) [2], which is in agreement with the results of a subsequent validation study (61%) [3]. This may be accounted by the higher rate of original ECDT criteria being fulfilled in MwA patients in our study compared to the development study (23 vs 3.9%) [2]. Patients with MwA may report symptoms of sudden onset, negative symptoms and/or lack of headache associated with aura, especially with advancing age [12, 17, 21]. Nevertheless, the specificity of the original EDCT criteria was low, when testing them against migraine aura with at least one atypical feature (i.e. symptom onset < 1 min). In context of the high specificity of the modified criteria, we argue that the clinical event as a whole, but not individual characteristics of symptoms are clinically relevant when differentiating an ischemic origin of symptoms from non-ischemic. Still, we have to stress that the misclassification of TIA without explicit diagnostic criteria would be worse than the data shown here. Despite some weaknesses of the EDCT in detecting migraine-like cerebral ischemic events, we believe they are a substantial improvement over a situation without such criteria. However, we would suggest that they should be used and further validated with a view to possible further improvement in future larger studies.

A migraine-like focal neurological symptom might be of ischemic, as well as of non-ischemic origin [20, 22]. The clinical differentiation of TIA from MwA traditionally shows a high rate of disagreement even between stroke-trained neurologists or in the setting of actor–patients providing a consistent history to different physicians [15]. Acknowledging that migraine aura-like symptoms can also be found in cerebral ischemia is, therefore, important from a clinical perspective and might improve the clinical assessment in the acute setting. Further, our data show that a purely stroke-like neurological event is likely not caused by a *typical* MwA. However, the value of the current scores in differentiating brain ischemia from *atypical* (i.e. probable [7]) migraine aura remains unclear. As the history of symptoms in some patients may not be discriminatory, the value of other clinical, serological or imaging markers might be necessary to improve accuracy of ischemia detection [13, 22, 23].

Our results are in-line with several previous studies. Eriksen et al. report sudden onset of visual aura and sensory symptoms in 19% and 25% of patients with migraine with aura [24]. Viana et al. found up to 37% of auras with multiple symptoms are characterized by two symptoms occurring simultaneously [21]. The same authors describe auras with negative visual and sensory symptoms [25] highlighting a variability of migraine aura which does not fully adhere to the canonical characterization of migraine aura [26]. On the other hand, patients with minor stroke frequently report atypical symptoms. In a retrospective review of a large stroke cohort, 20% had a progressive symptom onset and 11% had positive sensory symptoms [27]. In another large prospective study, progressive symptoms > 10 min were reported by 17% of patients with TIA or minor stroke [22]. These independent findings highlight the overlap and heterogeneity of symptom characteristics of migraine aura and ischemic stroke and explain, at least in part, our results.

Our study has strengths. We interviewed the stroke patients soon after the ischemic event, in order to avoid the recall bias. In patients with migraine, the recall bias is higher, but in our opinion, not significant since patients in general have experienced multiple migraine aura episodes during their lifetime. This is confirmed by the similar results after including only migraine patients interviewed within 1 week of the last migraine aura attack. Still, we cannot completely exclude a recall bias in patients with MwA and future prospective studies e.g. using diaries would be necessary to reduce this risk.

Our study also has limitations. We did not assess variability from aura to aura nor consistency of aura symptoms. We, however, assessed the most representative aura in each individual patient. In our study, we included patients with cerebral infarction to ensure that the diagnosis of ischemia is correct. This implies the limitation that we did not include patients with transient focal neurological symptoms of true ischemic origin with negative neuroimaging. However, the pathophysiology of a TIA is likely to be similar whether or not there is evidence of a lesion on neuroimaging. Approximately 40% of patients with TIA show an evidence of ischemic lesion on imaging [28, 29]. Predictors for ischemic lesion on neuroimaging are duration of symptoms of > 30 min, motor and speech disturbance but also time from symptom onset to imaging [28, 29]. The EDCT criteria are derived from characteristics observed at the *beginning* of the TIA, such as rapidity of symptom onset and simultaneous occurrence of two or more symptoms. Because a TIA onset is likely similar regardless of the presence of an ischemic lesion, we argue that the results of our study may be generalizable to TIA patients with negative neuroimaging.

Due to the low number of patients, we could not perform separate analyses on stroke patients with co-morbid migraine aura. It, therefore, remains unclear if an ischemic stroke in this group might more likely manifest with migraine aura-like symptoms, i.e. the value of the EDCT in these patients remains unclear [13, 30]. Lastly, the closed questions used in the questionnaire might have led to a higher proportion of atypical symptoms (e.g. symptom onset < 1 min) in the migraine group. However, they were created to reflect the way a patient is asked about symptoms in the clinical routine by general neurologists with the aim of avoiding suggestibility. For further improvement of the EDCT criteria, we looked for common stroke-related characteristics in stroke patients who do not fulfill the EDCT criteria (Supplemental Table). However, given the low number of patients, we found a high heterogeneity of data. Larger studies looking the characteristics of atypical stroke with migraine-like features are needed, to identify characteristics which might be helpful in further refining the EDCT.

In conclusion, the original and modified EDCT criteria have a sensitivity of 90% and 79%, respectively, for the detection of strokes in patients with transient neurological symptoms. Still, they represent a step forward in homogenizing the clinical diagnosis of ischemic attacks. In a minority of cases, they might be insufficient for excluding an ischemic mechanism in clinical routine. Due to the significant clinical impact of such misdiagnosis, further validation studies would be important to further improve diagnostic accuracy.

## Supplementary Information

Below is the link to the electronic supplementary material.

Supplementary file1 (DOC 99 KB)

Supplementary file2 (DOCX 15 KB)

## Data Availability

The data that support the findings of this study are available from the corresponding author upon reasonable request.
