# Supplementary material for: Field-testing the explicit diagnostic criteria for transient ischemic attack: a diagnostic accuracy study
Source: J Neurol. 2024 Dec 16;272(1):79. doi: 10.1007/s00415-024-12733-2 (PMC11649740; doi:10.1007/s00415-024-12733-2)
Supplement: Supplementary file 1 — Supplementary file1 (DOC 99 KB) [file 415_2024_12733_MOESM1_ESM.doc]

# ID

# Questionnaire

***If Stroke-Wake-Up?* □ yes □ no**

## 1) Have you had any visual disturbances? □ yes □ no. If yes,

### a) Type of visual disturbance?

□ blurred □ bright □ colored □dark/black □ zigzag lines □scintillating scotoma □ other

b) Localization: □monocular □ binocular homonymous □binocular on totality of visual field

c) Migration of visual disturbances? **□** yes □no (were fixed). If yes, please select:

□from center to periphery

if checked: □to the left □to the right □upwards □downwards

**□**From the periphery to the center

if checked: □ from above **□** from below □ from the right □ from the left

### e) Time of onset

□ suddenly □ < 59 seconds □60sec-4 minutes 59 seconds □5-59minutes □60 minutes-24h □>24h □ unclear

### f) Duration of the visual disturbance (beginning to end)

□ < 59 seconds □60sec-4 minutes 59 seconds □5-59minutes □60 minutes-24h □>24h □ unclear

2) Have you had any sensory disturbances? □yes □no.

a) Type of sensory disturbance? □tingling/pins and needles □numbness □ other

b) If yes, where were they located? (please select)

□ left - □ right - □ both sides

□ tongue - □ face - □ arm - □ leg □ torso

### c) Have they migrated? □yes □no

### If yes, please specify order (tongue, face, arm, leg, trunk)

### d) Time of onset

□ suddenly □ < 59 seconds □60sec-4 minutes 59 seconds □5-59minutes □60 minutes-24h □>24h □ unclear

### e) Duration of the sensory disturbance (beginning to end)

□ < 59 seconds □60sec-4 minutes 59 seconds □5-59minutes □60 minutes-24h □>24h □ unclear

### *3) Have you had any weakness?* □yes □no. If yes,

### a) where were they located? (please choose)

□ left - □ right - □ both sides

□ tongue - □ face - □ arm - □ leg - □torso

### b) Have they migrated? □yes □no

### If yes, please specify order (tongue, face, arm, leg, trunk)

### c) Time of onset

□ suddenly □ < 59 seconds □60sec-4 minutes 59 seconds □5-59minutes □60 minutes-24h □>24h □ unclear

### d) Duration of the motor disturbance (beginning to end)

□ < 59 seconds □60sec-4 minutes 59 seconds □5-59minutes □60 minutes-24h □>24h □ unclear

### 4) Have you had difficulty speaking? □yes □no.

If yes

### a) of what kind? (please choose)

□difficulty finding the right words □difficulty understanding what others have said □difficulty articulating the words □unfamiliar words spoken □neologisms □others

### b) Time of onset

□ suddenly □ < 59 seconds □60sec-4 minutes 59 seconds □5-59minutes □60 minutes-24h □>24h □ unclear

### c) Duration of the speech disturbance (beginning to end)

□ < 59 seconds □60sec-4 minutes 59 seconds □5-59minutes □60 minutes-24h □>24h □ unclear

### 5) a) What was the order of: Visual disturbances, speech disturbances, sensory disturbances, and weakness? Please describe:

### b) Time interval between symptoms? Eg, Sensory->Weakness->Visual disturbance

*Symptom 1 __________ ____after ______ minutes*

*Symptom 2_______________after_______minuets*

*Symptom 3_______________after_______minutes*

*Symptom 4_______________after_______minutes*

*Symptom 5_______________after_______minutes*

## 6) How many episodes with the symptoms described above have you had so far?

## when did the last episode occur?

## 7) Have you experienced a headache after or at the same time as the symptoms described above?

***If yes***

- *Did the symptoms* occur □ before □after, or □during the headache?

*- where was the headache located? __ ___________________*

- *Character* □pulsating pain □throbbing pain □shooting pain □pressing pain □burning

*- how long did it last? ________________*

*- how severe was the pain? (1-10/10) ______*

*- Was the pain related to physical activity?* □Yes □No

*if yes* □ the pain decreased □the pain increased

- during the headache you had □ nausea, □photophobia, □phonophobia

## 8) Have you had trouble concentrating? □yes □no.

If so,

### Duration (beginning to end)

□ < 59 seconds □60sec-4 minutes 59 seconds □5-59minutes □60 minutes-24h □>24h □ unclear

## 9) Were you been disoriented? □yes □no

If so,

### Duration of disorientation (onset to end)

□ < 59 seconds □60sec-4 minutes 59 seconds □5-59minutes □60 minutes-24h □>24h □ unclear

## 10) Have you had trouble swallowing? □yes □no

If yes

### Duration of difficulty swallowing (beginning to end)

□ < 59 seconds □60sec-4 minutes 59 seconds □5-59minutes □60 minutes-24h □>24h □ unclear

## 11) Have you had difficulty walking? □yes □no:

If so,

### a) Time of onset

□ suddenly □ < 59 seconds □60sec-4 minutes 59 seconds □5-59minutes □60 minutes-24h □>24h □ unclear

### b) Duration of the difficulty walking (beginning to end)

□ < 59 seconds □60sec-4 minutes 59 seconds □5-59minutes □60 minutes-24h □>24h □ unclear

## 12) Have you experienced trouble coordination in your arms (e.g., when spreading butter on bread) or legs (e.g., when walking)? □yes □no.

If so,

**a) where have you felt it?**

□ left - □ right - □ both sides

□ Tongue - □ Face - □ Arm - □ Leg - □Torso

### a) Time of onset

□ suddenly □ < 59 seconds □60sec-4 minutes 59 seconds □5-59minutes □60 minutes-24h □>24h □ unclear

### b) Duration of the coordination disturbance (beginning to end)

□ < 59 seconds □60sec-4 minutes 59 seconds □5-59minutes □60 minutes-24h □>24h □ unclear

## 13) Have you had vertigo? □yes □no

If so,

### a) was this

□rotatory to the right □ rotatory to the left □ swaying

### b) Time of onset

□ suddenly □ < 59 seconds □60sec-4 minutes 59 seconds □5-59minutes □60 minutes-24h □>24h □ unclear

### c) Duration of the vertigo (beginning to end)

□ < 59 seconds □60sec-4 minutes and 59 seconds □5-59minutes □60 minutes-24h □>24h □ unclear

### 14) Have you had double vision? □yes □no

...If so,

### a) were they □horizontal □vertical □ oblique offset? (please choose)

### b) Time of onset

□ suddenly □ < 59 seconds □60sec-4 minutes 59 seconds □5-59minutes □60 minutes-24h □>24h □ unclear

### c) Duration of the double vision(beginning to end)

□ < 59 seconds □60sec-4 minutes 59 seconds □5-59minutes □60 minutes-24h □>24h □ unclear

### 15) Have you had a hearing disturbance? □yes □no

If so,

### a) Time of propagation

□ < 59 seconds □60sec-4 minutes 59 seconds □5-59minutes □60 minutes-24h □>24h □ unclear

### b) Duration of the hearing disturbance (beginning to end)

□ < 59 seconds □60sec-4 minutes 59 seconds □5-59minutes □60 minutes-24h □>24h □ unclear

## 16) Cardiovascular risk factors

1. Do you smoke? If yes ______PY
2. Do you drink alcohol? If yes _________Drinks/month
3. Do you take drugs?
4. Have you been diagnosed with high blood pressure? □yes □no
5. Have you been diagnosed with diabetes mellitus? □yes **□**no
6. Do you have a family history of stroke and/or heart attack? **□**yes □no. If yes, at what age did these occur? _____
7. Do you have high cholesterol? **□**yes □no
8. Have you been diagnosed with sleep apnea? **□**yes □no □unknown
9. How much do you weigh and how tall are you? ____kg_____cm
10. Have you been diagnosed with depression? **□**yes □no
11. (for women) are you taking birth control pills? □yes □no
12. Have you been diagnosed with renal insufficiency? □yes □no
13. Have you been diagnosed with atrial fibrillation □yes □no, or other cardiac arrhythmias? If yes, which ones? _______
14. Have you been diagnosed with a chronic inflammatory disease? □ yes □ no

## 17) Varia

Have you been diagnosed with: peripheral arterial disease □, coronary heart disease□
