# Supplementary material for: Field-testing the explicit diagnostic criteria for transient ischemic attack: a diagnostic accuracy study
Source: J Neurol. 2024 Dec 16;272(1):79. doi: 10.1007/s00415-024-12733-2 (PMC11649740; doi:10.1007/s00415-024-12733-2)
Supplement: Supplementary file 2 — Supplementary file2 (DOCX 15 KB) [file 415_2024_12733_MOESM2_ESM.docx]

**Supplementary Table 1**: Characteristics of stroke patients who did not fulfill the modified EDCT criteria.*

| **Patient no** | **Stroke location** | **Stroke etiology** | **Modified EDCT criteria not fulfilled** |
| --- | --- | --- | --- |
| 1 | cerebellar in superior cerebellar artery and posterior inferior cerebellar artery territories | large artery atherosclerosis | C1, C3, C4 |
| 2 | pons | microvascular | C2, C3, C4 |
| 3 | gyrus pre-centralis | cardioembolic | C1, C2, C3, C4 |
| 4 | middle cerebral artery territory, basal ganglia | large artery atherosclerosis | C1, C2, C3, C4 |
| 5 | post-central gyrus | large artery atherosclerosis | C1, C2, C3 |
| 6 | thalamus, occipital and cerebellar | large artery atherosclerotic stenosis or cardioembolic atrial flutter | C2, C3, C4 |
| 7 | anterior choroid artery, basal ganglia | embolic stroke of undetermined source | C1, C2, C3, C4 |
| 8 | occipital lobe | iatrogenic after angiography for a posterior communicans artery aneurysm | C1, C2, C3, |
| 9 | thalamus | patent foramen ovale | C1, C2, C3 |
| 10 | watershed region between middle and posterior cerebral arteries | large artery atherosclerosis stenosis or cardioembolic atrial flutter | C1, C2, C3, C4 |
| 11 | postcentral gyrus | vertebral artery dissection | C1, C2, C3, C4 |

*None of the stroke patients fulfilled the modified EDCT criteria when not fulfilling the original EDCT.
